# Controversial Topics in Total Knee Arthroplasty: A Five-Year Update (Part 2)

**DOI:** 10.5435/JAAOSGlobal-D-19-00048

**Published:** 2020-01-06

**Authors:** Johannes Michiel van der Merwe, Matthew Semrau Mastel

**Affiliations:** From the Adult Reconstruction (Dr. van der Merwe), Department of Orthopaedics, University of Saskatchewan, and the Department of Orthopaedics (Dr. Mastell), University of Saskatchewan, Saskatoon, Saskatchewan, Canada.

## Abstract

This review article examines updates to the literature during the past 5 years on numerous topics which were felt to have ongoing controversy. These topics include venous thromboprophylaxis, tranexamic acid usage, tourniquet usage, and wound closure techniques.

**Methods:**

For each individual topic, a literature search was conducted on several databases with emphasis on studies that were published in the past 5 years. Preference was given to meta-analyses and randomized controlled trials.

**Results:**

Tranexamic acid is a safe and effective treatment modality, and consideration should be given to use multiple doses and combine different modes of administration. Certain treatment modalities (skin sutures, limited or no tourniquet usage) can cause greater patient satisfaction at a cost of longer operating times. Postoperative anticoagulation is still a very controversial topic. There is however some evidence suggesting prolonging anticoagulation to 35 days postoperative.

**Conclusions:**

By analyzing the results of the aforementioned studies, surgeons can implement the most up-to-date evidence-based care when doing total knee arthroplasty surgery. However, many of these selected topics continue to have a component of ongoing controversy with no definitive conclusions developed in recent literature.

Total knee arthroplasty (TKA) is a very commonly done orthopaedic procedure, and therefore, any improvements in technique may have a notable effect on the patient cohort. Despite the frequency of TKA, there are notable variations in techniques with many controversies existing. This review article examines updates to the literature during the past five years on numerous topics which were felt to have ongoing controversy or discrepancies between the techniques used by orthopaedic surgeons. In this article, attention is focused on venous thromboprophylaxis, tranexamic acid usage, tourniquet usage, and wound closure techniques. Venous thromboembolism is unfortunately a serious complication after TKA that is associated with notable patient morbidity, mortality, and economic cost.^1^ Thromboprophylaxis has been a topic of ongoing debate with literature and guidelines providing a variety of pharmacologic recommendations.^1^ With the introduction of tranexamic acid, the likelihood of a transfusion after TKA has dramatically decreased.^2,3^ Still, there is discrepancies regarding the most appropriate dosage, frequency, and preferred route of administration for tranexamic acid.^4,5,6,7^ Tourniquet usage during TKA has become routine to improve exposure and to enhance cementing techniques. However, some argue tourniquet use causes no difference in cement penetration and can be associated with residual thigh pain and quadriceps weakness.^8-10^ Wound closure is a critical aspect of TKA because it influences outcomes, patient satisfaction, and overall costs.^11^ Numerous closure materials exist including traditional sutures, barbed sutures, staples, and adhesives. In addition, there has been ongoing debate regarding the optimal position of the knee during closure for optimal soft-tissue repair and postoperative range of motion (ROM).

This review was not intended to be a comprehensive review of all these specific topics, but rather to be a compilation overview of updates to the literature from the past five years. By analyzing the results of recent studies, we can implement the most up-to-date and evidence-based care for our patients when doing TKA surgery.

## Methods

For this review, four topics were selected that were felt to have the most ongoing controversy among orthopaedic surgeons and in the literature. These topics are venous thromboprophylaxis, tranexamic acid usage, tourniquet usage, and wound closure techniques. For each individual topic, a literature search was conducted on several databases that included but was not limited to PubMed, the University of Saskatchewan Online Library Catalogue, *the Journal of the American Academy of Orthopaedic Surgeons* (*JAAOS*), and Google Scholar. Searches were conducted using a variety of search terms related to each specific topic in addition to the following terms: total knee arthroplasty, total knee replacement, total knee, and arthroplasty. As the focus of this study was to examine updates to the literature, emphasis was placed on studies that were published in the past 5 years (2014 to 2018 inclusive), with exceptions made in the case to provide background information on a topic, or studies that contain the most recently available data. Preference was given to meta-analyses, randomized controlled trials, and commonly used guideline sources, although other studies of lower level of evidence were included as required. Only articles and guidelines written in English and that were available in full-text format were included. For topics with numerous studies available from the past 5 years, the outcomes of each study were compiled into a table format.

## Venous Thromboprophylaxis

Venous thromboembolism (VTE) is a complication after TKA that is associated with notable patient morbidity, mortality, and economic cost. Thromboprophylaxis has been a topic of ongoing debate with literature and guidelines providing a variety of recommendations.^1^

Thromboprophylaxis can be divided into mechanical and pharmacological prevention. Mechanical methods include mobilization, graduated compression stockings, and intermittent pneumatic compression devices (IPCDs).^1^ IPCDs can have a relative risk reduction for deep venous thrombosis (DVT) and pulmonary embolism (PE) of greater than 50%, without the risk of bleeding, or any other notable complication.^12^ However, compliance remains a major challenge limiting their use.^12^ Given the possible effectiveness combined with the low-risk profile, the American College of Chest Physicians (ACCP) recommends dual prophylaxis with an antithrombotic agent and an IPCD during the hospital stay (grade 2C).^12^

## Pharmacologic Methods

Pharmacological methods of prophylaxis include unfractionated heparin (UFH), low-molecular-weight heparin (LMWH), vitamin K antagonists (VKAs), synthetic pentasaccharide factor Xa inhibitors (fondaparinux), newer oral anticoagulants (ie rivaroxaban, dabigatran, and apixaban), and aspirin. The most appropriate agent, along with duration of therapy, has been the focus of notable attention in TKA literature. There are many guidelines published (Table 1), although discrepancies continue to persist mainly because of the lack of evidence supporting one particular agent or regimen. The latest 2012 evidence-based guidelines from the American College of Chest Physicians (ACCP) provide suggestions based on efficacy while balancing safety that can assist with choosing a prophylactic regimen.^12^ Although they maintain a preference for LMWH based on its long-term safety data,^12^ rivaroxaban and aspirin have become more popular agents.

**Table 1:** Select ACCP, NICE, Thrombosis Canada, SIGN, and AAOS Recommendations for VTE Prophylaxis in Patients Undergoing TKA

Guidelines	Recommendations	Duration of Prophylaxis
ACCP (2012)^12^	Preference: LMWH Other acceptable options: low-dose UFH, VKA, fondaparinux, apixaban, dabigatran, rivaroxaban,aspirin, or IPCD Recommends dual prophylaxis with addition of intermittent pneumatic device (IPCD) during the hospital stay	Minimum 10-14 days but suggest extending up to 35 days
NICE^20^	Choose one of: Aspirin (75 or 150 mg) LMWH + antiembolism stockings (until D/C) Rivaroxaban Consider apixaban or dabigatran if the above cannot be used	14 days 14 days 14 days
Thrombosis Canada^21^	Rivaroxaban (10 mg PO daily) Apixaban (2.5 mg PO BID) Dabigatran (220 mg PO daily) Enoxaparin (30 mg SC BID or 40 mg SC daily) Dalteparin (5000 U SC daily) Tinzaparin (4500 U or 75 U/kg SC daily) Fondaparinux (2.5 mg SC daily) Nadroparin (38 U/kg SC daily (day 1-3 postop), 57U/kg SC daily (day 4+) Patients not at high VTE risk: consider rivaroxaban until postoperative day 5 then ASA 81 mg daily for 9 additional days ASA alone and VKAs not included given accessibility of more effective and equally or more convenient alternatives	14-35 days Suggest longer duration for patients at greater risk including bilateral TKA, previous VTE, and substantially impaired mobility at discharge
SIGN^22^	Acceptable options (combined with mechanical prophylaxis unless contraindicated): LMWH,fondaparinux, rivaroxaban, and dabigatran Aspirin NOT recommended as sole pharmacological agent	Optimal duration unclear Extended prophylaxis should be given
AAOS^23^	Unable to recommend for or against specific prophylactics Current evidence unclear about which prophylactic strategy or strategies is/are optimal or suboptimal	Patients and physicians discuss the duration of prophylaxis due to lack of reliable evidence

AAOS = American Academy of Orthopaedic Surgeons; ACCP = American College of Chest Physicians; BID = twice daily; LMWH = low-molecular-weight heparin; PO = orally; SIGN = Scottish Intercollegiate Guidelines Network, NICE = National Institute for Health and Care Excellence; TKA = total knee arthroplasty; UFH = unfractionated heparin; VKA = vitamin K antagonist; VTE = Venous thromboembolism; D/C = patient discharge; SC = subcutaneous injection

### 

#### Unfractionated Heparin

UFH is a mixture of glycosaminoglycans that inactivates thrombin, factor Xa, along with other coagulation enzymes. It is not absorbed orally so must be given parenterally. The ACCP estimates the effect of low-dose UFH to be a reduction of 13 symptomatic VTEs per 1000 patients undergoing major orthopaedic surgery.^12^ Major risks include major bleeding and heparin-induced thrombocytopenia (HIT).^1^

#### Low-Molecular-Weight Heparin

LMWH is formed by depolymerization of UFH. It has greater activity against factor Xa than thrombin and has more predictable pharmacokinetic and pharmacodynamic properties than UFH.^1^ Longer half-life results in less frequent parenteral dosing.^1^ The risk of HIT is still present but reduced.^1^ The ACCP considers LMWH the benchmark to compare other agents against and makes the suggestion to use LMWH over other agents based mainly on its longer safety record.^12^ Although the synthetic pentasaccharide fondaparinux has greater activity against factor Xa than LMWH, has a greater half life, and does not cause HIT, it is still less appealing for DVT prophylaxis because of its adverse events^1^ (i.e. major bleeding requiring reoperation 4 more per 1000 compared with LMWH).^12^

#### Vitamin K Antagonists

VKAs (ie, warfarin) are a group of oral anticoagulants that interfere with the cyclic interconversion of vitamin K and its 2,3 epoxide. The use of VKAs requires ongoing monitoring, and the benefit of its use is closely balanced by a possible increase of major bleeding.^1,12^ Although the latest ACCP guidelines recommend VKAs as an acceptable option,^1^ the delays in achieving therapeutic effect, as well as interactions with other medications and dietary changes, make it a less desirable option.^13^

#### Novel Oral Anticoagulants

The major advantage of the new oral anticoagulants is the ability to be administered orally at regular intervals and without the need for monitoring. Rivaroxaban and apixaban are direct factor Xa inhibitors. Rivaroxaban was more effective for VTE prevention than enoxaparin after TKA in phase III clinical trials.^1^ However, it has an increased risk of bleeding.^12^ Apixaban has similar efficacy and comparable risk of major bleeding as LMWH.^12^ Dabigatran is a reversible, direct thrombin inhibitor and was found to be as effective as enoxaparin for VTE prophylaxis, with a similar propensity to cause bleeding.^1^ The latest ACCP guidelines recommend all three of these agents, although they suggest the greater long-term experience with LMWH still favors its use.^1^

Ma et al in a meta-analysis that included 13,790 TKA patients found the overall incidence of DVT to be significantly less with the use of rivaroxaban (relative risk [RR] = 0.59, *P* < 0.01) and apixaban (RR = 0.60, *P* < 0.01) compared with enoxaparin, although there was no significant difference in the rates of PE. In addition, there was no significant difference in the rates of major bleeding.^14^

Guang-Zhi et al in a meta-analysis that included nine trials and 15,829 patients with total hip arthroplasty (THA) and TKA similarly reported that compared with enoxaparin, rivaroxaban had significantly lower rates of symptomatic DVT (RR = 0.36, *P* = 0.0001), but not the rate of symptomatic PE (RR = 0.79, *P* = 0.57). However, they suggested that rivaroxaban was associated with a significant increased risk of major bleeding (RR = 1.37, *P* = 0.02), but was not different in terms of all-cause mortality (RR = 0.63, *P* = 0.27). Ultimately, they suggested more evidence is needed to verify the risk of major bleeding with rivaroxaban.^15^

Kapoor et al in a network meta-analysis including 94 studies analyzed the efficacy of VTE prophylaxis, and safety in avoiding hemorrhage, in 12 different prophylactic strategies. Relative to LMWH, direct oral Xa inhibits had markedly lower rates of DVT (odds ratio [OR] 0.45; 95% confidence interval (95% CI) 0.35 to 0.57). Aspirin was done similarly to LMWH, while VKA predicted 56% more DVT events.

When including only symptomatic DVTs, direct factor Xa inhibitors led to 4-fold fewer symptomatic DVTs (OR 0.25, 0.13 to 0.47), while small study numbers and event rates limited the conclusions of all other strategies. Compared with LMWH, direct oral Xa inhibitors had increased rates of major bleeding (OR 1.21, 95% CI 0.79 to 1.90), although not statistically significant. VKA and aspirin performed equally to LMWH in terms of efficacy and major bleeding. Overall, they suggest direct oral Xa inhibitors have the most desirable profile when considering efficacy and avoidance of major bleeding, while VKA has an unfavorable profile and aspirin an indeterminate profile.^16^

#### Aspirin

The use of aspirin has increased since the latest ACCP guideline (2012) considered it an acceptable agent. It is less effective at VTE prophylaxis than other agents, but may be associated with a lower risk of bleeding.^17^ This change in ACCP recommendations was based on the Pulmonary Embolism Prevention Trial which involved 4088 elective hip and knee arthroplasty patients and 13,356 hip fracture patients with a 36% reduction in symptomatic DVT rate among the hip fracture group.^17^ There was no observed difference in rates between aspirin and placebo in the arthroplasty group, and overall, there was a trend toward more major bleeding with aspirin.^17^ However, the ACCP suggested the data were sufficient to recommend aspirin as a prophylactic option compared with no prophylaxis.^12,17^

Drescher et al in a systematic review and meta-analysis studied 8 trials with 1408 patients after hip fracture surgery, THA, and TKA. When comparing aspirin to all other anticoagulants (studies were included that compared aspirin against VKA, UFH, LMWH, thrombin inhibitors, pentasaccharides, and factor Xa/IIa inhibitors dosed for VTE prevention), they found there was no difference in rates of proximal DVT after knee or hip arthroplasty (9.3% versus 9.7%, RR: 1.00; 95% CI: 0.49 to 2.05). The absolute number of PEs was higher in the aspirin group, although the frequency was low, and the difference was not statistically significant. The difference in rates of all bleeding events (aspirin 3.9%, anticoagulants 7.8%), as well as major bleeding (aspirin 2.1% versus anticoagulants 0.6%), was not statistically significant between aspirin and anticoagulants for the lower extremity arthroplasty group. They concluded the balance between VTE and bleeding risks suggests that aspirin may be favorable after elective TKA.^18^

An et al^19^ have done a systematic review and meta-analysis involving 69,551 THAs and TKAs. The overall rate of VTE was low (DVT 1.2%, PE 0.6%) when aspirin was used as prophylaxis, with the rate of major bleeding only 0.3%.^19^ However, they found that the quality of evidence was limited.^19^

Hybrid strategies have also emerged with numerous regimens suggested. Anderson et al^13^ performed a multicenter, double-blind, randomized controlled trial (RCT) of 3424 patients undergoing THA or TKA either randomized to continuation of rivaroxaban or changed to aspirin 81 mg after an initial 5-day course of rivaroxaban. There was no significant difference in prevention of symptomatic VTE (0.64% ASA, 0.70% rivaroxaban; *P* < 0.001 for noninferiority) or major bleeding (0.47% aspirin, 0.29% rivaroxaban; *P* = 0.42).^13^

The low price and accessibility, along with oral administration, make aspirin an enticing choice, although its use for VTE prophylaxis still remains controversial. Although the ACCP (2012) and NICE (2018) guidelines include aspirin as an acceptable option,^12,20^ Thrombosis Canada (2018) did not include aspirin alone as a suggested option,^21^ the SIGN (2015) guidelines continue to recommend against aspirin as a sole agent given the greater effectiveness of other agents,^22^ and the Efort 2018 Review “remains skeptical about the use of aspirin as a sole method of prophylaxis.”^1^

#### Venous Thromboprophylaxis Summary

Choosing the appropriate method of prophylaxis for your patient requires a thorough knowledge of the available agents, specifically their efficacy and risk profiles. There are many guidelines published (Table 1),^12,20,21,22,23^ and the ACCP provides a thorough review based on efficacy and risk.^12^ The ACCP continues to suggest tinzaparin over other agents based on its long-term risk data^12^; however, many studies have shown direct factor Xa inhibitors such as rivaroxaban to have an equivalent or greater reduction in symptomatic VTE rates, with a similar risk profile.^1,12,14-16^ The use of aspirin has become increasingly popular, with numerous studies suggesting a similar efficacy in VTE prevention compared with anticoagulants, along with a favorable risk profile.^12,13,18-20^ However, the use of aspirin for VTE prophylaxis does still remain controversial, and although its use is supported by several guidelines,^12,20^ others recommend against it, and additional investigation would be beneficial before making firm conclusions.^1,21,22^ The ACCP recommends consideration be given to extend prophylaxis up to 35 days, rather than only 10 to 14 days,^12^ and the Thrombosis Canada guidelines also recommend a duration of 14 to 35 days with a longer duration for those patients at higher risk.^21^ Based on ACCP guidelines, dual prophylaxis with the addition of IPCDs while in hospital should be done.^12^

## Tranexamic Acid Usage

TKA is a major surgery associated with blood loss, which could lead to a transfusion postoperatively. Allogeneic transfusions do have risks to the patient, which could lead to an inferior result. These risks include increased infection rate, metabolite abnormalities, hemolysis, and blood-borne illnesses. With the introduction of tranexamic acid, the likelihood of a transfusion has dramatically decreased.^2,3^ Still, there is some controversy regarding the dosage, frequency, and preferred route of administration for tranexamic acid. Certainly, when adding a new medication such as tranexamic acid, one has to be certain that there will not be resultant adverse events such as deep vein thrombosis or pulmonary embolism. By focusing on randomized controlled trials over the past 5 years, we attempted to answer these aforementioned questions.

There are three main routes that tranexamic acid is administered. They are as follows: intravenously at weight adjusted doses ranging from (10–20) mg/kg 2 hours preoperatively; orally with 2 g dispensed 2 hours preoperatively; and intra-articularly by injecting 1 to 3 g mixed with 20–100 cc of normal saline into the joint once the arthrotomy has been closed.

After administering the first oral or intravenous tranexamic acid dose, multiple subsequent doses can be given (oral dose: 1 g; intravenous dose: 10 mg/kg). This can include one to four additional doses given at time intervals varying from 3 hours, 6 hours, 9 hours, 12 hours, and 15 hours after surgery. Oral administration can follow an initial intravenous dose; however, the opposite order of administration does not usually occur.^4^

When we look at all the evidence accumulated over the past 5 years, there are no clear answers as to which route, dosage, combination of drugs, and frequency are the best. However, conclusions can be made with the evidence at hand. Comparing oral administration to intravenous administration, there appears to be no difference in outcomes.^2^ This seems to be similar comparing intravenous administration to intra-articular administration,^3,24,25^ as well as oral administration matching to intra-articular administration.

When evaluating the ideal frequency, there are many studies demonstrating benefit with multiple subsequent dosages compared with a single dose.^4,5,7^ The total number of doses and the frequency of the dosages still remain unclear with some studies showing clear benefit administering tranexamic acid at 3-hour intervals for four subsequent doses,^5^ while other studies show no benefit of adding even a third additional dose.^4^ The latter spaced the doses 6 hours apart (3 hours, 9 hours, and 15 hours postoperatively).

Combining routes, such as administering an intravenous dose followed by oral dosing or intravenous followed by intra-articular dosing, does seem to offer better results.^6,7^

Tranexamic acid does not appear to lead to higher morbidity as seen in multiple studies.^2,3,4,5,6,7,24,25^ However, patients excluded from these studies included previous deep vein thrombosis or pulmonary embolism in the past 6 months, serious cardiac or cerebrovascular issues, preoperative hepatic or renal dysfunction, bilateral procedures, and coagulation disorders^4,5^; therefore, it is difficult to know whether tranexamic acid is safe in all patients.

### 

#### In summary

Tranexamic acid is a crucial addition in total knee replacements. It has been proven to be safe and effective in reducing blood loss, without notable risk to the patient. Using oral tranexamic acid is a cheap alternative to the more expensive intra-articular and intravenous administrations. Consideration should be given to using multiple doses (at least two) in the acute postoperative period along with combining different routes of administration.^2,3,4,5,6,7,24,25^

## Tourniquet Usage

Tourniquet usage during TKA has become routine to improve exposure and to enhance cementing techniques. Multiple studies have been cited to demonstrate poor cement penetration if the cement is contaminated with circulating blood or tissue products.^8^ Alternatively, numerous studies have demonstrated no difference in cement penetration during cementation whether a tourniquet was used or not.^8-10^ It has also been shown that using a tourniquet for a prolonged period can cause quadriceps weakness, residual pain in the thigh, excessive inflammation, and muscle damage.^8-10^

The tourniquet can be inflated during multiple stages of the TKA including at the time of skin incision or just prior to cementing. Deflation can occur once the cement hardens or once the dressing and compressive bandage has been applied. Table 2 shows recent evidence regarding tourniquet use in respect to differences in blood loss, surgical time, postoperative pain, and ROM. It has been shown that intraoperative blood loss and operating time are markedly reduced when using a tourniquet during a TKA. However, total blood loss was similar whether the tourniquet was inflated at the beginning of the case or just before cementing. This can be explained by an increase in postoperative blood loss in the tourniquet group. Greater care is taken to obtain hemostasis intraoperatively in the no tourniquet (NT) or limited tourniquet (LT) groups compared with the bloodless field in the tourniquet group, which subsequently leads to less postoperative blood loss.^26-28^ This can also explain why the total operating time is increased in the NT and LT groups. Multiple studies demonstrated stronger quadriceps strength and better ROM in the NT and LT groups.^9,26,27,29^ NT or LT use also leads to less swelling and postoperative pain.^9,26,27,29,30^ This can be explained by the decrease in inflammation seen in the musculature with LT or NT usage.

**Table 2:** Recent Studies Comparing Outcomes Between Total Knee Arthroplasties Done With a Tourniquet (Inflation Once Skin Incision Is Made) (T), No Tourniquet (NT), and Limited Tourniquet Use (Inflation Once Cementing) (LT)

Study Comparison	Type of Study	Patients	Total Blood Loss	Total Surgical Time	Postoperative Pain	Range of Motion	Length of Stay
Zhou et al^26^—NT versus T	Prospective double-blinded RCT	150	No difference	Better in the T group (0.038)	Better in the NT group (*P* < 0.001)	Better in the NT group (*P* = 0.005)	Better in the NT group (P = 0.001)
Liu et al^30^—NT versus T	Prospective RCT	52	Not reported	Better in the T group (*P* < 0.05)	Better in the NT group (*P* < 0.05)	No difference	Not reported
Wang et al^9^—LT versus T	Observer blinded RCT	50	Better in the T group (*P*: 0.0411)	Better in the T group (*P*: 0.0449)	Better in the LT group	Better in the LT group	Not reported
Harsten et al^31^—T versus NT	RCT	64	Not reported	Not reported	No difference	No difference	No difference
Fan et al^27^—T versus LT	RCT	60	No difference	No difference	Better in the LT group (*P* < 0.001)	Better in the LT group (*P* < 0.001).	Not reported
Ejaz et al^29^—T versus NT	RCT	70	Not reported	No difference	Better in the NT group (*P*: 0.02)	Better in the NT group	Not reported
Tarwala et al^28^—LT versus T	RCT	71	No difference	No difference	No difference	No difference	Not reported

### 

#### In summary

The results of tourniquet usage are still very controversial. There is, however, a tendency to less pain, better ROM, and increased quadriceps strength with no tourniquet or limited tourniquet usage at a cost of longer operating times.^9,26,27,29,30^ These aforementioned benefits are mainly evident in the first several weeks after TKA.^9,26,27,28,29,30,31^ In seeking the ultimate pain strategy after TKA to attempt to improve outcomes, no tourniquet or limited tourniquet usage could form part of the holistic approach. Although it may result in slightly longer surgical times,^9,26,30^ it is a simple intervention which could lead to better patient outcomes with very minimal risk, including no difference in total blood loss.

### Wound Closure

Wound closure is a critical aspect of TKA as it influences outcomes, patient satisfaction, and overall costs. Suboptimal closure can lead to higher rates of wound complications including superficial and deep infections, abscess formation, dehiscence, and poor cosmesis.^11^ The ideal wound closure should optimize healing and reduce infection while producing a satisfactory scar. Closure techniques that aim to decrease surgical time, along with complications, will ultimately reduce total costs as well.^11^ Numerous closure materials exist including traditional sutures, barbed sutures, staples, and adhesives.

Sutures are the traditional method for deep and superficial closure and continue to be one of the most commonly used methods. Sutures reliably provide adequate closure at the cost of increased surgical time.^11^

**Figure 1:**
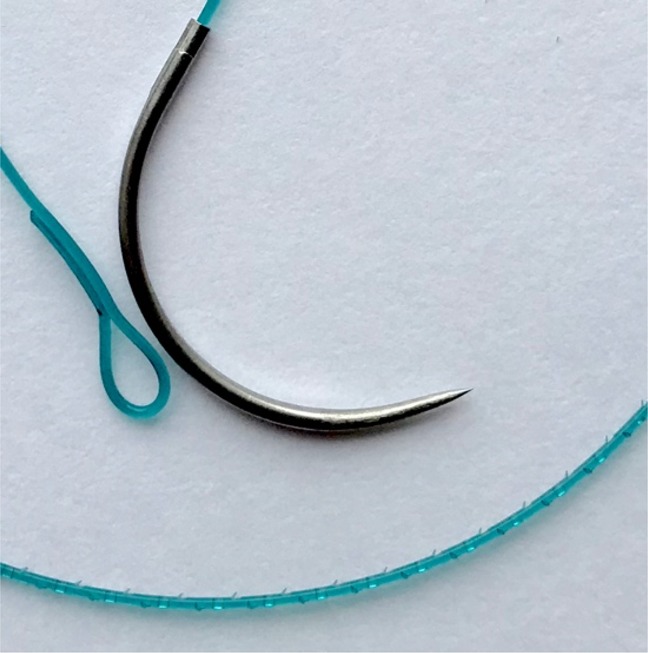
Photograph of Covidien V-Loc 180 suture demonstrating an example of unidirectional barbed suture.

More recently barbed sutures (Figure 1) have gained popularity for both superficial and deep wound closure. These sutures have unidirectional or bidirectional barbs, eliminating the need for knots. This can lead to faster application, and it has been suggested that they are more resistant to failure due to multiple points of adherence.^11^

Stainless steel staples along with traditional sutures are the most common methods of superficial closure. Staples can be efficiently inserted and removed, although removal can cause some patient discomfort.^11^

Adhesives can be used as a substitute to or in addition to cutaneous sutures. When used as a substitute, the subcutaneous tissue is often reinforced with greater amounts of suture which may negate any time saved by using the adhesive.^11^ Adhesives are less commonly used than sutures or staples and will not be focused on in this review.

The optimal technique for wound closure is still debatable with different materials being available for both superficial and deep closure, each with advantages and disadvantages. There have been numerous studies comparing the options while focusing on variables such as wound complications, closure time, overall costs, and cosmesis (Table 3).^11,32,33,34,35^

**Table 3:** Comparison of Recent Studies Evaluating Traditional Sutures (TS), Barbed Sutures (BS), and Staples (S) for Wound Closure in TKA

Study Comparison	Wound Complications	Closure Time	Cost	Cosmesis	Recommendation
Krebs et al^11^ (evidence-based review 2018) (TS) versus (BS) versus (S)	No notable difference (4 studies) Superior blood flow with subcuticular (TS) versus (S)(2 studies)	Deep: faster with (BS) versus (TS) (5 studies) Superficial: faster (S) versus (TS) (4 studies) (S) versus (BS) more study needed	Potential savings with (BS) due to faster surgical time and fewer resources used	No difference (TS) versus (S) (2 studies)	No optimal closure technique developed More studies needed Deep closure: (BS) fastest with similar complications (TS) Superficial closure: (S) fastest, (TS) may have improved blood flow/healing, more studies necessary to evaluate (BS) for superficial layers
Kim et al^32^ (meta-analysis and systematic review)—8 studies, 828 TKAs (TS) versus (S) for superficial closure	Favored (S) for both deep and superficial infection although not statistically significant	Faster with (S)	Overall reduction in resource utilization with (S) Eggers et al^33^ total intraoperative costs: $1023.50 USD (TS) and $636.70 USD (S)	Not reported	(S) may have subtle clinical advantages over (TS) for superficial closure More study needed
Zhang et al^34^ (systematic review and meta-analysis) 9 articles, 1729 patients (BS) versus (TS)	No difference *P* = 0.38	(BS) 3.56 minutes faster than (TS) (*P* < 0.01)	(BS) = average $290.72 USD savings over (TS) (*P* < 0.01)	Not reported	(BS) leads to shorter OR times + decreased costs over (TS) Suggest (BS) is optimal method for closure of arthrotomy, subcutaneous, and subcuticular layers
Campbell et al^35^—retrospective review—416 pts 247 (S), 129 (BS) (BS) versus (TS) superficial closure	Superficial infection: 3.2% (S), 11.8% (BS); *P* = 0.001 Deep infection: 0.8% (S), 4.7% (BS); *P* = 0.018 Dehiscence: 1.2% (S), 4.1% (BS) *P* = 0.098	Not reported	Not reported	Not reported	(BS) should be avoided for superficial closure

TKA = total knee arthroplasty

### 

#### Deep Closure

For deep closure, the traditional method has been interrupted sutures, although there has been an increasing use of barbed sutures. Krebs et al^11^ suggested deep closure was fastest with barbed sutures with no difference in complications or cosmesis compared with traditional sutures. The resultant decrease in resources and faster surgical times also have potential for cost savings. Ultimately, the authors suggested more studies were needed to make firm recommendations. A meta-analysis by Zhang et al^34^ also found no difference in complications, along with improved time and cost savings, and suggested barbed sutures were the optimal method for closure of the arthrotomy, subcutaneous, and subcuticular layers.

#### Superficial Closure

For superficial closure, sutures were again the traditional method and continue to be one of the most common methods along with staples. In the aforementioned review by Krebs et al,^11^ they suggested staples decrease surgical time over sutures, while sutures may have improved tissue blood flow and healing. They did not note any notable differences between the two options in terms of wound complications or cosmesis. Kim et al also suggested that staples may have a subtle advantage over sutures.^32^ Although statistically insignificant, the rates of superficial and deep infection, as well as wound dehiscence, favored staples. In addition, staples had improved surgical times with a resultant decrease in overall resource utilization.^32^ However, both of these articles suggested more studies are necessary.^11,32^ More recently barbed sutures have also been introduced for superficial closure, although evidence is more limited.^34^ In the meta-analysis by Zhang, they suggested that decreased surgical time, overall cost, with no difference in complications makes barbed sutures the optimal method for superficial closure over traditional suture.^34^ This is in stark contrast to Solovyova et al. who suggested in their mini-review that when used for subcuticular or skin closure, there are several studies that show increased wound complications with barbed suture.^36^ Campbell et al^35^ also demonstrated increased rates of both superficial and deep infection after using barbed suture for superficial closure. Several studies have demonstrated that barbed suture is more efficient than traditional suture and has no difference in complication rates when used for arthrotomy closure.^11,34^ However, as several studies show increased rates of wound complications, barbed sutures may not be the optimal choice for superficial closure at this point until further evidence emerges.^35,36^

## 

### Knee Position for Closure

There has been ongoing debate regarding the optimal position of the knee during closure for optimal soft-tissue repair and postoperative ROM. King et al^37^ initially described closing the knee in deep flexion (90 to 120°) to cause less stretching of soft tissues potentially leading to less patient discomfort, along with preventing extensor mechanism shortening with resultant increased tension with deep knee flexion. Evidence in the past has been limited, and therefore, the method of closing the wound has been based on surgeon preference.^38^ More recent literature (Table 4) is still indeterminate with minimal differences noted in long-term flexion ROM, function, or complications.^38-40^ However, Faour et al^39^ in a review of seven studies including 516 patients noted improved early ROM, faster functional recovery, comparable satisfaction, and no higher risk with wound closure in flexion; therefore, suggested closure in flexion may have a potential advantage compared with closure in extension. However, more evidence is required to make definitive conclusions, and the choice can be made based on surgeon preference.

### 

#### In summary:

For deep closure (arthrotomy), current evidence suggests both traditional and barbed sutures have similar rates of complications and overall cosmesis.^11^ However, improved surgical times with barbed sutures, along with a resultant decrease in overall costs, may provide a slight advantage over traditional suture.^11,34^ For superficial closure, sutures and staples are both acceptable options with similar rates of wound complications and cosmetic outcomes.^11,32^ However, improved surgical times and resultant costs may favor staples, although with increased patient discomfort at removal.^11,32^ The evidence for superficial barbed sutures is mixed, likely making them a less desirable option than staples or sutures.^34-36^ Based on the current evidence, there is no notable difference in long-term outcomes between wound closure with the knee in flexion or extension, although it has been suggested that there may be some benefit to the patient in the early recovery period with closing the knee in flexion.^38-40^ However, as more evidence is required, the decision can continue to be made based on surgeon preference.

**Table 4:** Comparison of Recent Studies Evaluating Wound Closure in Extension (E) Versus Flexion (F)

Study Comparison	Post-Operative ROM	Function	Complications	Recommendation
Faour et al^13^ (2018) Literature Review—7 studies, 516 patients (259 [F], 257 [E])	Improved early ROM with (F) (4 positive,.3 neutral studies) No difference with long-term ROM recovery	Improved early postop pain scores (2 positive, 1 neutral study), faster functional recovery (2 studies) with (F) No difference long-term recovery, pain scores, knee function (KSS-5 neutral studies), or satisfaction	No difference in wound-related complications (seven neutral studies)	May have improved early ROM, faster functional recovery, comparable satisfaction, and no higher risk with wound closure in flexion; therefore, closure in (F) may have potential advantage compared with (E)
Cerciello et al^39^ (2016) systematic review—6 articles, 397 TKAs	Variable at time of closure (60 to 110° [F]) No difference at final f/u (avg. 8 months)(101.7° [F] and 102.4° [E]; *P* > 0.05)	No differences at final f/u: KSS (45.8 [F], 48.2 [E]), AKSS (20.2 [F], 20.8 [E]), VAS (1.2 [F], 1.1 [E])	No differences	No clear advantage to capsule closure in (F) or (E) Decision based on surgeon preference
Motififard et al^40^ (2016) Double-blind prospective RCT, 85 patients-44 (E), 41 (F)	No differences After 1 week, 2 weeks, 4 weeks, 6 months, or 12 months (*P* < 0.05)	No differences KSS after 12 months: Knee score 71.8 ± 10.9 (F), 74.3 ± 11.5 (E) Function Score 68.3 ± 12.1 (F), 68.4 ± 14.7 (E)	Not reported	Wound closure position does not affect postop flexion ROM or KSS

TKA = total knee arthroplasty

## Summary

There are numerous controversial topics in total knee arthroplasty and four of these areas were addressed in this particular review article. Despite the evolution of literature specific to these topics, there continues to be an amount of uncertainty regarding the most beneficial and appropriate treatment choices. Based on this review, Tranexamic acid is a safe and effective treatment to reduce blood loss. Consideration should be given to use multiple doses of tranexamic acid and to combine different modes of administration. Limited tourniquet use or no tourniquet use do show a tendency to better patient satisfaction at a cost of longer operating times. Barbed sutures might be beneficial in deep arthrotomy closure, but staples or sutures are still recommended for skin closure. There may be some benefit to close the knee in flexion. The evidence is clear that we should use postoperative anticoagulation. Thromboprophylaxis should be individualized to the patient, and anticoagulation administered with a thorough knowledge of their efficacy and risk profiles. Consideration should be given to extend anticoagulation to 35 days postoperatively. The conclusions that we arrived at were based on studies done in the past five years and are certainly not a comprehensive overview of each of the topics. Orthopaedic surgeons should use these recommendations cautiously and only as an adjunct to their existing knowledge about the topic. There has been an abundance of literature published in the past five years attempting to address these problems with total knee arthroplasty, and future studies will only continue to improve the functional outcomes and satisfaction of patients.
